# The Development of an UAV Borne Direct Georeferenced Photogrammetric Platform for Ground Control Point Free Applications

**DOI:** 10.3390/s120709161

**Published:** 2012-07-04

**Authors:** Kai-Wei Chiang, Meng-Lun Tsai, Chien-Hsun Chu

**Affiliations:** Department of Geomatics, National Cheng-Kung University, No.1, University Road, Tainan 701, Taiwan; E-Mails: kwchiang@mail.ncku.edu.tw (K.-W.C.); chienhsun0229@msn.com (C.-H.C.)

**Keywords:** direct georeferencing, INS, GPS, UAV

## Abstract

To facilitate applications such as environment detection or disaster monitoring, the development of rapid low cost systems for collecting near real time spatial information is very critical. Rapid spatial information collection has become an emerging trend for remote sensing and mapping applications. In this study, a fixed-wing Unmanned Aerial Vehicle (UAV)-based spatial information acquisition platform that can operate in Ground Control Point (GCP) free environments is developed and evaluated. The proposed UAV based photogrammetric platform has a Direct Georeferencing (DG) module that includes a low cost Micro Electro Mechanical Systems (MEMS) Inertial Navigation System (INS)/Global Positioning System (GPS) integrated system. The DG module is able to provide GPS single frequency carrier phase measurements for differential processing to obtain sufficient positioning accuracy. All necessary calibration procedures are implemented. Ultimately, a flight test is performed to verify the positioning accuracy in DG mode without using GCPs. The preliminary results of positioning accuracy in DG mode illustrate that horizontal positioning accuracies in the x and y axes are around 5 m at 300 m flight height above the ground. The positioning accuracy of the z axis is below 10 m. Therefore, the proposed platform is relatively safe and inexpensive for collecting critical spatial information for urgent response such as disaster relief and assessment applications where GCPs are not available.

## 1. Introduction

**Figure 1.**
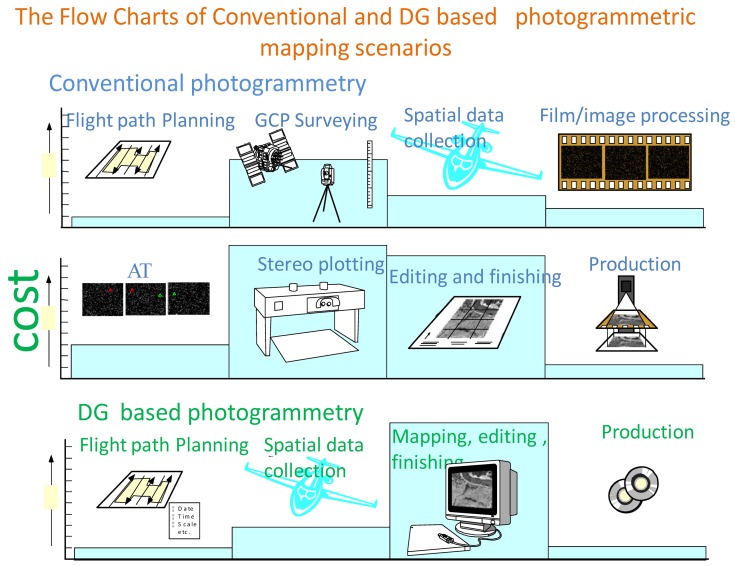
Procedures applied for conventional aerial photogrammetry (adopted from [1]).

With the number of disasters increasing due to climate change, the development of a rapidly deployable and low cost system to collect near real time spatial information has become very critical. Therefore, the rapid spatial information acquisition capability has become an emerging trend for remote sensing and mapping applications. Airborne remote sensing, more specifically aerial photogrammetry, in its classical form of film-based optical sensors (analogue) has been widely used for high accuracy mapping applications at all scales and rapid spatial information collection for decades. Recently, the film-based optical sensors (analogue) have been replaced by digital imaging sensors. Figure 1 depicts the procedures applied for conventional aerial photogrammetry.

In general, conventional photogrammetric operation methods rely on GCPs. Although photogrammetry has adopted digital technology, GCPs are generally considered the only source of reliable georeferencing information. Recently, DG technology has become possible by integrating GPS and INS, making exterior orientation parameters available with sufficient accuracy at any instant of time [2]. The integration of INS/GPS improves the georeferencing of photogrammetric data and frees it from operational restrictions. Together with digital data recording and data processing, it allows multi-sensor systems.

Operational flexibility is greatly enhanced in all cases where a block structure is not needed [2]. Costs are considerably reduced, especially in areas where little or no ground control is available. Current achievable accuracy of commercial DG systems is sufficient for many mapping applications. As shown in Figure 1, the cost and production efficiency have significantly improved with the use of DG based photogrammetric platforms.

However, there are some limitations for current DG based photogrammetric platforms. The cost of renting a plane to conduct aerial photogrammetry is high and there are strict regulations and complicated procedures for obtaining a permit to conduct a flight plan in most countries. In addition, the flexibility and capability of conducting small area surveys or rapid spatial information collection is rather limited.

Therefore, a DG based airborne platform that is relatively free of government regulations and relatively inexpensive but maintains high mobility for small area surveys or rapid spatial information acquisition is desired for urgent response such as disaster relief and assessment.

On the other hand, the Integrated Sensor Orientation (ISO) combining benefits from both DG and in-direct georeferencing with traditional bundle adjustment using Aerial Triangular (AT) process can be considered a practical and robust process for modern airborne photogrammetry applications when the imagery is flown in a block configuration with sufficient overlap [3]. By using the Exterior Orientation Parameters (EOP) provided by DG systems as initial approximates for bundle adjustment, only a limited number of tied points in overlapping area is needed. GCPs are applied to check datum shift and compensate for residual systematic errors of differential GPS [3]. Generally speaking, its advantages include combining advantages from DG and traditional AT process as well as the possibility to use less accurate Inertial Measurement Unit (IMU), however, its primary limitation for potential disaster applications is the requirement of block imagery [3], which might not be always available for disaster relief applications. Therefore, the ISO is not implemented in this study. Table 1 shows the comparison of various sensor orientation methods.

Numerous studies have been conducted on the application of UAV for photogrammetry applications. Despite the availability of very high resolution satellite imagery, large scale photogrammetric mapping is still performed primarily with aerial images. The reason is that satellite images have several constrains such as weather, availability of stereo coverage, temporal and geometric resolution, minimum area order, and price. Thus, airborne platforms such as aircraft, helicopter, kite, balloon, and UAVs are a very good and generally inexpensive alternative. Moreover, the latest developments of small and medium format digital cameras are remarkable with corresponding advances in automated image processing. For large areas, aircraft are usually employed as a platform for acquiring aerial images. For small and remote area mapping, UAVs are a very good and inexpensive platform and imaging alternative, in particular in developing countries.

The main applications of UAVs are observation, maintenance, surveillance, monitoring, remote sensing and security tasks [4]. In recent years, an increasing number of UAV based photogrammetric platforms have been developed, with their performances proven in certain scenarios [5].

A detailed review of UAV photogrammetric applications can be found in [6,7]. Although most schemes apply low cost INS/GPS integrated systems for flight control, a DG based UAV photogrammetric platform equipped with an INS/GPS integrated Positioning and Orientation System (POS) that can provide exterior orientation parameters of the camera in a GCP free environment has not been proposed until recently. Nagai *et al.* [8] first proposed a UAV borne mapping system using a UAV helicopter as the platform, equipped with an INS/GPS POS to facilitate DG capability. GPS and IMU data are processed with the Kalman filter (KF). In addition, the bundle adjustment of Charge-Coupled-Devices (CCD) images is made with the support of INS/GPS integrated POS solutions. The INS/GPS integrated POS solutions and results of bundle block adjustment are then fused to generate positioning and orientation data for further processing [8]. Eisenbeiss [9] used a UAV with a state-of-the-art navigation and control system to accurately produce of dense Digital Surface Models (DSM), three-dimensional vector maps, and high resolution orthophotos. Grenzdorffer *et al.* [10] monitored agricultural and forestry areas with a micro-UAV.

Table 2 compares various photogrammetric platforms in terms of system configuration and applications. Generally speaking, the selection of a platform is application dependent. The primary objective of developing a UAV based photogrammetric platform is to meet requirements such as small operational area, rapid deployment, low cost, high mobility and acceptable positioning accuracy. Therefore, it is not practical to use those platforms as replacements for conventional photogrammetric applications [1].

Most current platforms that apply a conventional bundle adjustment process or AT with GPS require a large number of GCPs. Therefore, the objective of this study is to develop a DG based UAV photogrammetric platform primarily for GCP free applications. An INS/GPS integrated POS system is implemented to provide DG capability for the proposed platform. Instead of using the KF for optimal estimation, a backward smoother is implemented to enhance the accuracy of the POS onboard. In addition, most current commercially available UAV photogrammetric platforms apply GPS pseudorange measurements in Single Point Positioning (SPP) mode to determine the trajectory for conventional bundle adjustments. In contrast, the POS module developed in this study applies GPS L1 carrier phase measurements to be processed in differential mode. The kinematic positioning accuracies of the proposed POS module in SPP mode with pseudorange measurements and differential mode with L1 carrier phase measurements are 5 m and 1 m, respectively [1].

## 2. Technical Configurations of Proposed Platform

The components of each module are described in detail in the following sections.

### 2.1. Specifications of Proposed UAV Photogrammetric Platform

**Figure 2.**
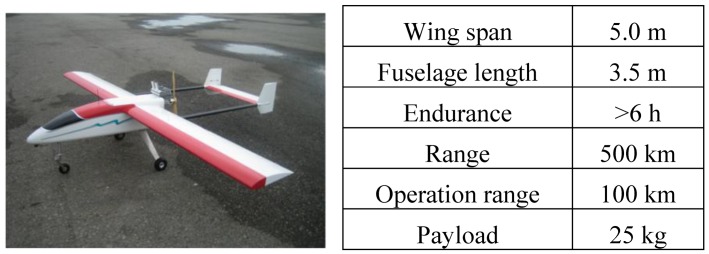
Proposed UAV platform.

The proposed UAV platform and its specifications are illustrated in Figure 2. As shown in the figure, the proposed UAV is designed for medium range applications. The wing span is 5 m and the payload is 25 kg. The maximum operational range is 100 km and the real time video transmission range is 100 km with extended range communication links. The flexible flight altitude and six hours endurance time make it suitable for small area and large scale photogrammetric missions.

### 2.2. Configuration of DG Module

**Figure 3.**
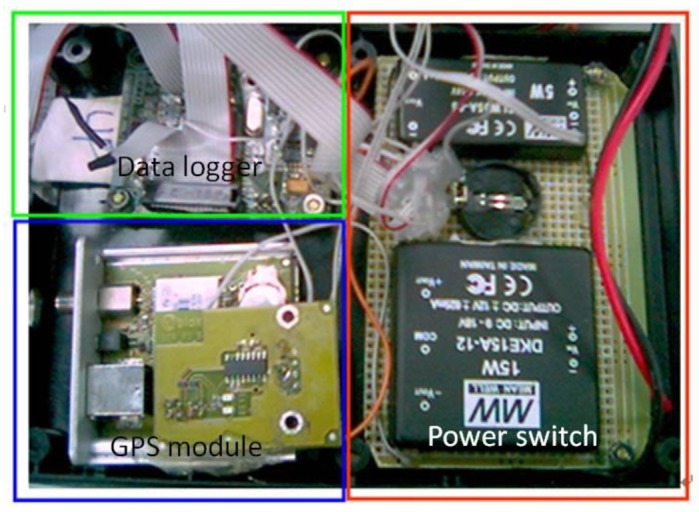
Configuration of DG module.

Figure 3 shows the DG module designed in this study for facilitating GCP free photogrammetry applications and INS/GPS POS aided bundle adjustment photogrammetry. The GPS receiver (EVK-6T from U-blox) is applied in the DG module. This model is chosen because it can provide L1 carrier phase raw measurements to be applied for differential GPS processing with single frequency carrier phase measurements to provide sufficient positioning accuracy. It addition, it supplies Pulse Per Second (PPS) output used to synchronize the time mark used to trigger the camera in the DG module.

The IMU used for the DG module is MMQ-G from BEI SDID. This model is chosen due to its compact size and weight. The MMQ-G IMU integrates MEMS quartz rate sensors (100 deg/h in run bias) and vibrating quartz accelerometers. The total budget of proposed POS module is around 10,000 US dollars. To supply the power required for the individual sensors with various power requirements from the battery, a power switch module was designed. An RS232 port is implemented to transmit the measurements collected by the MMQ-G IMU applied to the data storage module. Since EOS 5D Mark II has its own power supply, it is not considered in the power supply design. The data storage module used to record the measurements collected by MMQ-G, EVK-6T, and the synchronized time mark used to trigger the Canon 5D Mark II is Antilog from Martelec. Due to the limitations of the payload and power supply, a PC or notebook based data storage module is ruled out in this study. A simple mechanization that can store measurements communicated though a serial port is thus required.

**Figure 4.**
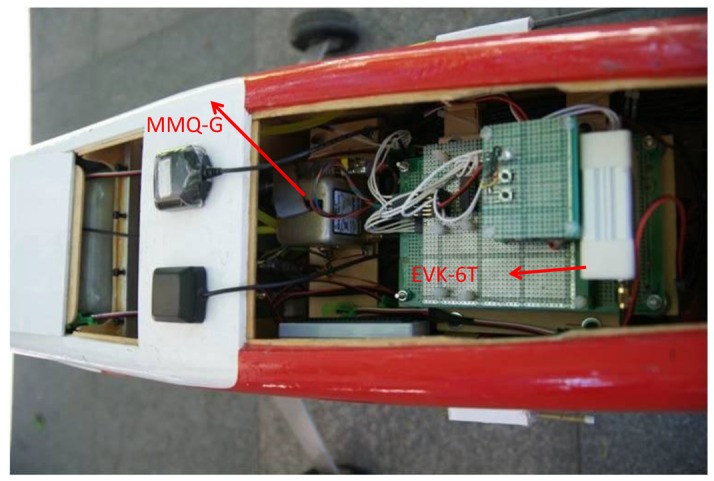
Set up of DG module in UAV.

The Antilog is chosen due to its unrivalled flexibility, low power consumption, and reliability. Since EOS 5D Mark II has its own storage mechanization, it is not included in this module. Figure 4 illustrates the set up of DG module within the UAV platform. As shown in the figure, the MMQ-G IMU is on the top of the camera. The nominal location of the GPS antenna is on the body of the airplane, which is marked permanently.

**Figure 5.**
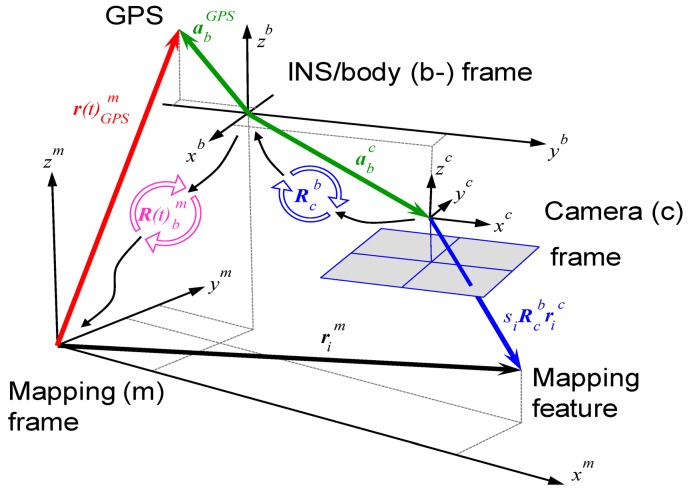
Concept of airborne DG.

Equation (1) and Figure 5 illustrate the general concept of airborne DG. With this implementation, the coordinates of a mapping feature can be obtained directly through measured image coordinates. However, this procedure works based on the *a priori* knowledge of various systematic parameters, as shown in the following expression:

(1) $${\text{r}}_{\text{i}}^{\text{m}}=\text{r}{\left(\text{t}\right)}_{\text{nav}}^{\text{m}}+{\text{R}(\text{t})}_{\text{b}}^{\text{m}}({\text{S}}_{\text{i}}{\text{R}}_{\text{c}}^{\text{b}}+{\text{r}}_{\text{i}}^{\text{c}}+{\text{r}}_{\text{ins}}^{\text{c}}-{\text{r}}_{\text{ins}}^{\text{GPS}})$$

where ${\text{r}}_{\text{i}}^{\text{m}}$ is the coordinate vector of point (i) in the mapping frame (m-frame), ${\text{r}}_{\text{nav}}^{\text{m}}(\text{t})$ is the interpolated coordinate vector of the navigation sensors (INS/GPS) in the m-frame. **S^i^** is a scale factor, determined by stereo techniques, laser scanners or DTM, ${\text{R}}_{\text{b}}^{\text{m}}(\text{t})$ is the interpolated rotation matrix between the navigation sensor body frame (b-frame) and the m-frame, (t) is the time of exposure, *i.e.*, the time of capturing the images, determined by synchronization, ${\text{R}}_{\text{c}}^{\text{b}}$ is the differential rotation between the C-frame and the b-frame, determined by calibration, **r^c^** is the coordinate vector of the point in the C-frame (*i.e.*, image coordinate), ${\text{r}}_{\text{INS}}^{\text{c}}$ is the vector between IMU center and camera principal point, determined by calibration, and ${\text{r}}_{\text{INS}}^{\text{GPS}}$ is the vector between IMU center and GPS antenna center, determined by calibration.

**Figure 6.**
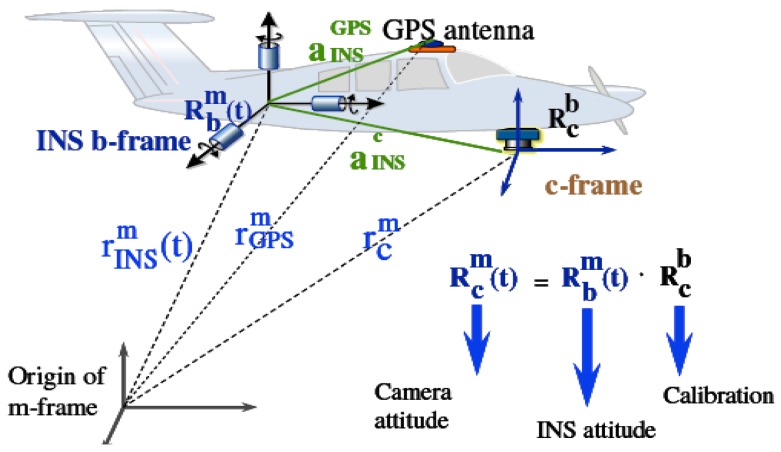
Concept of boresight angle calibration.

**Figure 7.**
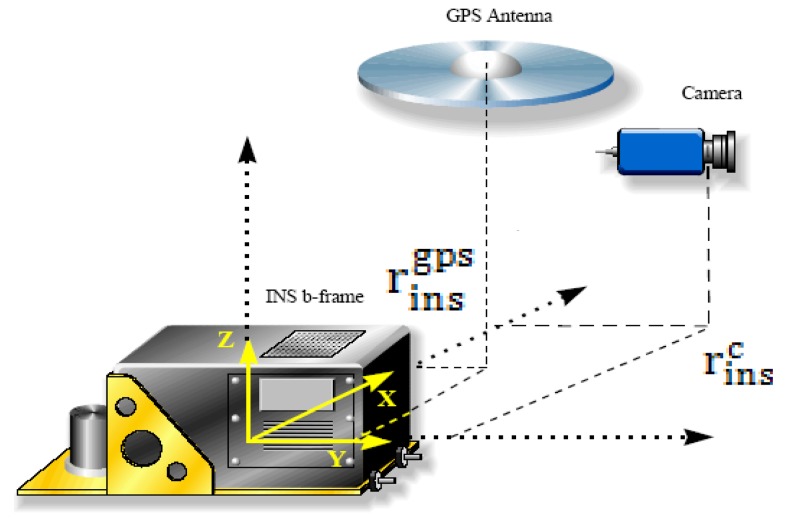
Concept of lever arm calibration.

The physical meanings of ${\text{R}}_{\text{c}}^{\text{b}}$, ${\text{r}}_{\text{ins}}^{\text{c}}$, and ${\text{r}}_{\text{ins}}^{\text{gps}}$ are given in Figures 6 and 7, respectively. A two-step boresight angle calibration procedure is implemented in this study to acquire the rotation matrix $\left({\text{R}}_{\text{c}}^{\text{b}}\right)$ between the camera and IMU by using the rotation matrix $\left({\text{R}}_{\text{b}}^{\text{m}}\right)$ provided by the IMU and the rotation matrix $\left({\text{R}}_{\text{c}}^{\text{m}}\right)$ provided by conventional bundle adjustment during the calibration procedure using the following equation [11]:

(2) $${\text{R}}_{\text{b}}^{\text{c}}={\text{R}}_{\text{m}}^{\text{c}}{\left({\text{R}}_{\text{m}}^{\text{b}}\right)}^{\text{T}}$$

**Figure 8.**
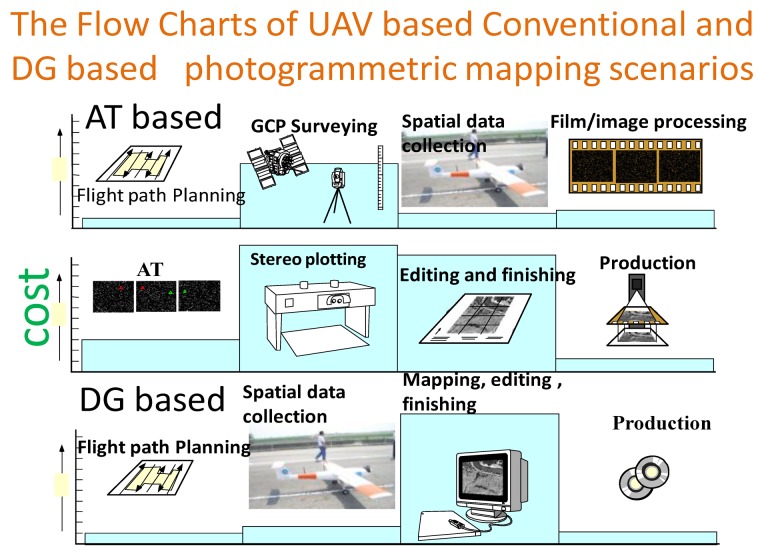
Procedures for UAV based photogrammetric procedure (adopted from [1]).

The lever arm ${\text{r}}_{\text{ins}}^{\text{gps}}$ between the GPS phase center and IMU center is determined through a surveying process. The lever arm ${\text{r}}_{\text{b}}^{\text{c}}$ between the camera and IMU centers is determined through a two-step procedure that compares the output of conventional bundle adjustment and INS/GPS integrated POS solutions during calibration process using the following equation:

(3) $${\text{r}}_{\text{c}}^{\text{b}}={\text{R}}_{\text{m}}^{\text{b}}\left(\begin{array}{c} {\text{X}}_{\text{b}}^{\text{m}}-{\text{X}}_{\text{c}}^{\text{m}}\\ {\text{Y}}_{\text{b}}^{\text{m}}-{\text{Y}}_{\text{c}}^{\text{m}}\\ {\text{Z}}_{\text{b}}^{\text{m}}-{\text{Z}}_{\text{c}}^{\text{m}} \end{array}\right)$$

where ${\text{r}}_{\text{c}}^{\text{b}}$ illustrate the lever arm vector to be estimated, $\left({\text{X}}_{\text{b}}^{\text{m}},{\text{Y}}_{\text{b}}^{\text{m}},{\text{Z}}_{\text{b}}^{\text{m}}\right)$ represent the positional vector of INS center in the mapping frame provided by INS/GPS integrated POS solutions and $\left({\text{X}}_{\text{c}}^{\text{m}},{\text{Y}}_{\text{c}}^{\text{m}},{\text{Z}}_{\text{c}}^{\text{m}}\right)$ represent the positional vector of camera center in the mapping frame provided by bundle adjustment. Once those parameters are well calibrated and the sensors are fixed on the platform, the proposed platform is able to conduct GCP free DG missions without conventional bundle adjustments for future flight thus the efficiency is improved significantly as conventional bundle adjustments is time consuming. INS/GPS integrated POS solutions can also be applied to assist conventional bundle adjustments [12]. Figure 8 shows the procedures and cost estimates of the proposed UAV based photogrammetric platform. Similar to Figure 1, conventional AT based and DG based procedures are proposed, respectively. Generally speaking, the spatial data collection expanse with UAV DG operation is lower by 50%∼60% compared to commercial airplane with DG operation shown in Figure 1 [1].

As shown in Figures 1 and 8, the proposed DG based UAV photogrammetric can significantly reduce the cost of field work including aerial photography and GCP surveys. On the other hand, lab work might increase due to the increase of images to be processed. Generally speaking, the proposed platform is not aimed to be a replacement of conventional DG based photogrammetry; the aim is to fill the gap when it is not convenient or practical to perform rapid spatial information acquisition missions in small areas with conventional DG based photogrammetry [6,7].

## 3. Data Processing Strategy

For the determination of the lever arm and boresight angle parameters, the EOP must be solved by the close-range bundle adjustment. However, some errors are introduced during the image measurements due to the manufacture imperfection of cameras. Thus, camera calibration must be performed. The objective of camera calibration is to analyze the Interior Orientation Parameters (IOP) such as the lens distortion, focal length, and principle point. These systematic errors can be diminished during the image point measurements. For system calibration and DG measurements, a camera control field and a ground control field were established.

### 3.1. Camera Indoor Calibration Field

**Figure 9.**
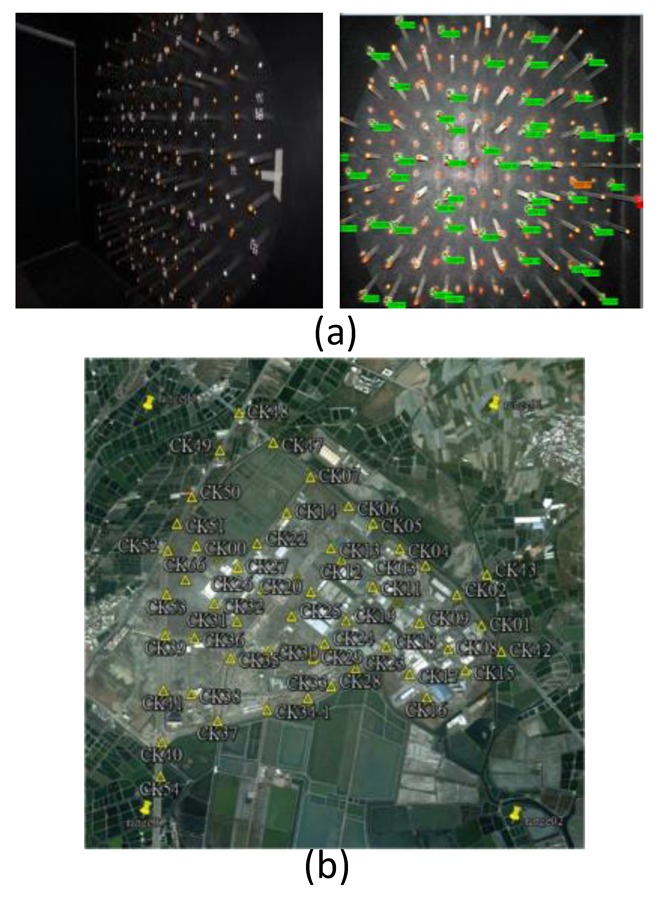
(**a**) Indoor calibration field; (**b**) Distribution of GCPs in test field.

Figure 9(a) shows the indoor calibration field applied in this study to calibrate the IOP of Canon EOS 5D Mark II.

Because the digital CCD camera is rather than the traditional camera which can use the flame frame to rectify the systematic error and image coordinates measurement, a bundle method with self-calibration is proposed for determining the IOP of the camera [11]. Those obtained IOP are applied to enhance the accuracy of EOP estimation and the DG task. Figure 9(b) shows the distribution of GCP which are set up every 400 m in the test field. The GCPs are accurately surveyed using Real Time Kinematic (RTK) GPS surveying technique and processed with network adjustment software. The standard deviation of the GCPs is around 3 mm thus they are applied to calibrate the lever arms and boresight angles.

### 3.2. Ground Control Field

In this research, a two-step approach is implemented to conduct the lever arm and the boresight angle calibrations. The image acquisition for the calibration process was performed by flying the UAV photogrammetric platform over the ground control field with 300 m flight height above ground. The measurements of the image points were processed. The Australis software was then used to calculate the EOP of the images through bundle adjustment. After performing the interpolation of INS/GPS positioning and orientation parameters at the image exposure time, the differences of the position and the orientation between the EOP acquired by a conventional photogrammetry procedure and interpolated INS/GPS positioning and orientation parameters were derived for further processing.

The perspective position of each image $\left({\text{r}}_{\text{c}}^{\text{m}}\right)$ was exactly known after applying the bundle adjustment then to be applied for lever arm calibration. The calculation of the INS/GPS position vector $\left({\text{r}}_{\text{ins}/\text{gps}}^{\text{m}}\right)$ at exposure time was conducted using interpolation. Then, the lever arm $\left({\text{r}}_{\text{ins}/\text{gps}\_\text{c}}^{\text{b}}\right)$ was solved using the following equation:

(4) $$\;{\text{r}}_{\text{ins}/\text{gps}\_\text{c}}^{\text{b}}={\text{R}}_{\text{b}}^{\text{m}}({\text{r}}_{\text{c}}^{\text{m}}-{\text{r}}_{\text{ins}/\text{gps}}^{\text{m}})$$

For boresight angle calibration, the rotation matrix between the camera frame and the mapping frame of each image $\left({\text{R}}_{\text{c}}^{\text{m}}\right)$ was obtained from the bundle adjustment results, and the rotation matrix between the body frame and mapping frame of each image $\left({\text{R}}_{\text{b}}^{\text{m}}\right)$ was measured by INS. The relationship is shown in Figures 6 and 7, respectively. Detailed numerical results of calibration are presented in the next section.

**Figure 10.**
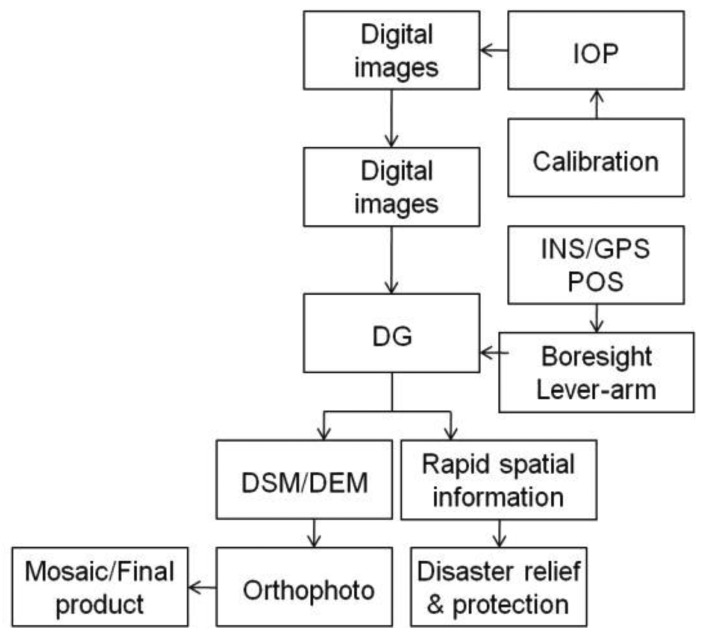
Proposed DG-ready photogrammetric procedure.

In theory, the lever arm and boresight rotation matrices derived from each image are the same; however, this is not exactly true in practice. Reasonable values from the calibration can be determined using appropriate weights or the average distribution. After obtaining the calibration parameters, the DG task can be performed without using any GCP. Figure 10 illustrates the DG based photogrammetric process proposed in this study.

### 3.3. Integrated POS Data Processing

Post-mission processing, when compared to real-time filtering, has the advantage of having the data of the whole mission for estimating the trajectory [13]. This is not possible when using filtering because only part of the data is available at each trajectory point, except the last. When filtering is used in the first step, an optimal smoothing method, such as the Rauch-Tung-Striebel (RTS) backward smoother, can be applied [14,15]. It uses the filtered results and their covariances as a first approximation. This approximation is improved by using additional data that was not used in the filtering process. Depending on the type of data used, the improvement obtained by optimal smoothing can be considerable [16].

**Figure 11.**
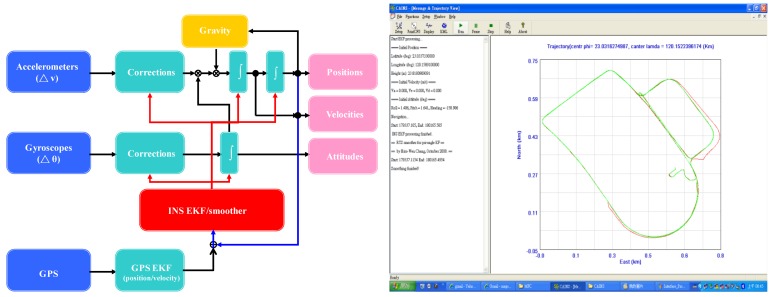
Loosely coupled INS/GPS integrated scheme.

For a georeferencing process which puts POS stamps on images and a measurement process that obtains three-dimensional coordinates of all important features and stores them in a Geographic Information System (GIS) database, only post-mission processing can be implemented due to the complexity [17]. Therefore, most commercially available DG systems operate in real time only for data acquisition and conduct most of the data processing and analysis in post-mission mode. Figure 11 shows the loosely coupled INS/GPS integrated scheme and processing engine implemented in this study.

## 4. Results and Discussion

**Figure 12.**
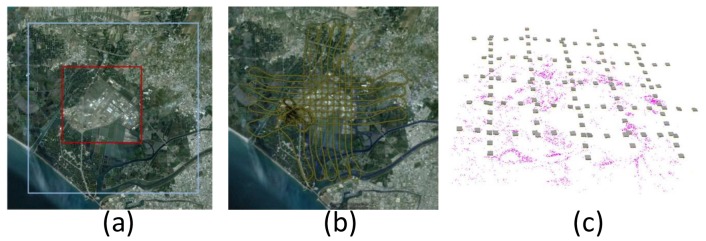
(**a**) Test area; (**b**) Trajectories of test flight (**c**) The distribution of tie points.

To validate the performance of the proposed platform, a field test was conducted in the fall of 2011. The area of the test zone was 3 km × 3 km, which is covered by the red square shown in Figure 12(a). The blue region indicates the fly zone approved for this test.

### 4.1. Flight Planning

The flight altitude for aerial photography was set to 300 m above ground. Owing to the limit of the payload and the impact of side wind affecting the attitude of the UAV, the endlap and sidelap were increased to 80% and 40%, respectively, to insure that the coverage of the stereo pair overlapped completely during the test flight. Although more images have to be processed, completed coverage of the stereo pair is guaranteed. Figure 12(b,c) illustrate the flight path and estimated coordinates for the camera exposure center along the trajectory.

### 4.2. Calibration Results

The camera calibration process was implemented to obtain the IOP of the camera, as mentioned in the previous section. Then, the lever arm and boresight angle were calibrated after installing the cameras on the UAV. Consequently, the performance analysis of DG accuracy was performed by comparing DG results with check points with precisely known coordinates. Table 3 shows the preliminary IOP results. The error of the camera calibration is acceptable at this stage, and may be improved in future work.

**Figure 13.**
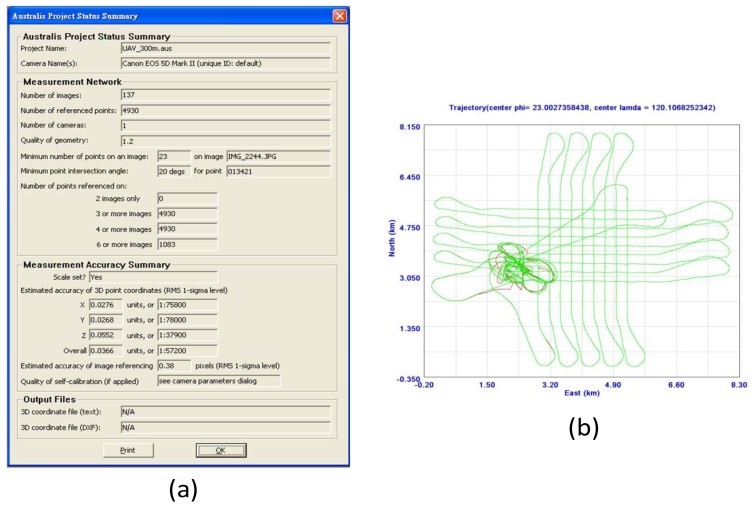
(**a**) EOP results; (**b**) Trajectory of integrated POS.

Figure 13(a) shows the EOP results. The estimated accuracy of image referencing is 0.38 pixels. The influence of the EOP is around 0.04 m in terms of the three-dimensional positioning accuracy. Figure 13(b) shows the trajectory of INS/GPS integrated POS solutions during the test. The INS/GPS integrated POS solutions processed with the Extended KF (EKF) trajectory is shown in red line and those processed with the RTS smoother are shown in green. Because of the kinematic alignment process applied due to the use of a low cost INS/GPS integrated POS module, the beginning (around 300 s) of the EKF trajectory is not smooth. However, only the smoothed trajectory (green) is applied for further processing.

A two-step approach was implemented to acquire the lever arm and boresight angle of each camera. First, the EOPs of the images were calculated through bundle adjustment by measuring the image points when the flight mission had completed. Second, the interpolation of INS/GPS smoothed POS solutions at the image exposure time was implemented. The lever arm and boresight angle were obtained by comparing the differences of the position and the attitude between the EOP and the interpolated INS/GPS solutions. Tables 4 and 5 show the lever arm and boresight angle results, respectively.

The Y axis of the IMU body frame, shown in Table 4, is the flight direction, the X axis is the lateral direction, and Z axis points upward, respectively. The lever arm parameters estimated by the proposed algorithm are reasonable in all directions as the IMU is placed on top of the camera. Similarly, the precisions (standard deviations) of those estimated boresight angles are reasonable based on the quality of the IMU applied. The quality of lever arm and boresight angles is highly correlated with flying height and dynamic. The calibration flight applied in this study is 300 m above around and quality can be improved by at least 50% by reducing the flying height to 150 m. However, a fixed wing UAV with flying height lower than 200 m might suffer stall issue thus it is not applied in this study. Therefore, the lever arm and boresight angles of the applied low cost POS module should be conducted in static mode instead of the kinematic mode applied in this study to achieve higher accuracy for further processing in the future.

### 4.3. Verification of DG Capability of Proposed UAV Photogrammetric Platform

**Figure 14.**
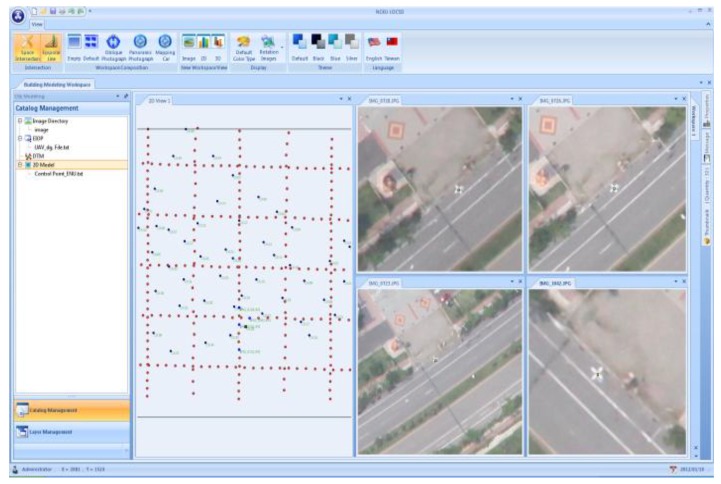
DG program.

The DG module, written in Visual Studio 2008 C++, is applied to calculate the coordinates of the check points. As shown in Figure 14, the coordinates of the control points, IOP, and EOP derived from INS/GPS POS solutions are imported into the software. The users can perform image point measurements on different images for a given feature. The results of the space intersection are obtained from various images that have common points of interest.

**Figure 15.**
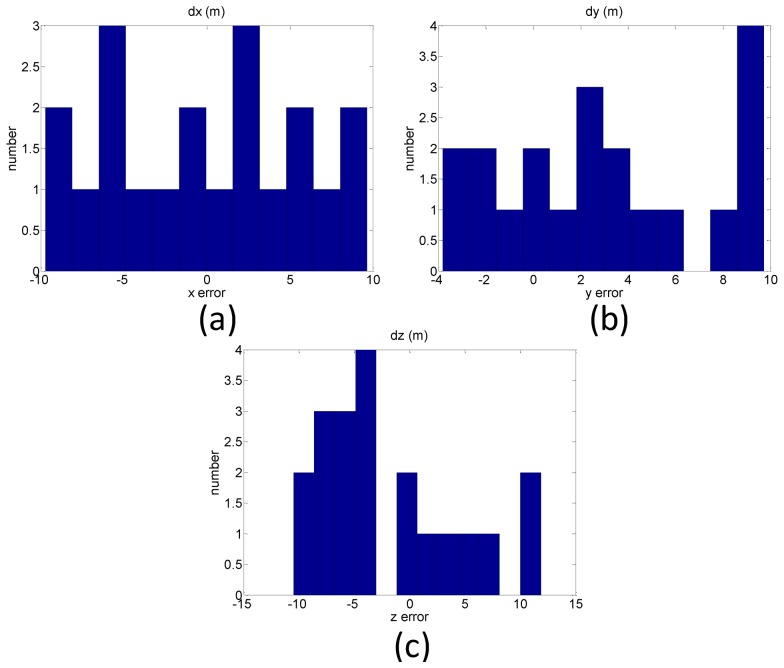
Positional error distribution in (**a**) x direction; (**b**) y direction; (**c**) z directions.

The reference coordinates of the check points are obtained through the precise control survey with GPS RTK technology and network adjustment software, as mentioned previously. The DG coordinates of the check points are then compared with their reference coordinates and their results are given in Table 6. Figures 15 shows the error distribution of the DG test compared with each check point in different direction. The Root Mean Square (RMS) errors in the x, y, and z axes are 5.71, 5.30, and 6.66 m, respectively. As shown in Figure 15, the percentage of the error distribution between ±5 m in x and y directions are 70% and 75%, respectively. On the other hand, the percentage of the error distribution between ±10 m in z direction is 85%. Most current commercial UAV photogrammetric platforms apply GPS SPP results to assist the AT procedure. This study uses GPS L1 carrier phase raw measurements, which can be applied for differential GPS processing with single frequency carrier phase measurements and increase the positioning accuracy from 2∼5 m to 1∼2 m for civilian purposes such as mapping and disaster monitoring.

Such specifications can be applied for the different scenarios to make orthophoto and three- dimensional vector maps. In addition, operators can get the position of each point on a photograph quickly for rescue operations. If a UAV flies over the same place periodically, changes in the flight area can be detected. DG results can be applied to analyze the place, area, and range changes in terrain appear. The main objective of this UAV study is to develop a low cost aerial platform capable of autonomous flight that is equipped with a photogrammetric payload for rapid mapping purposes.

**Figure 16.**
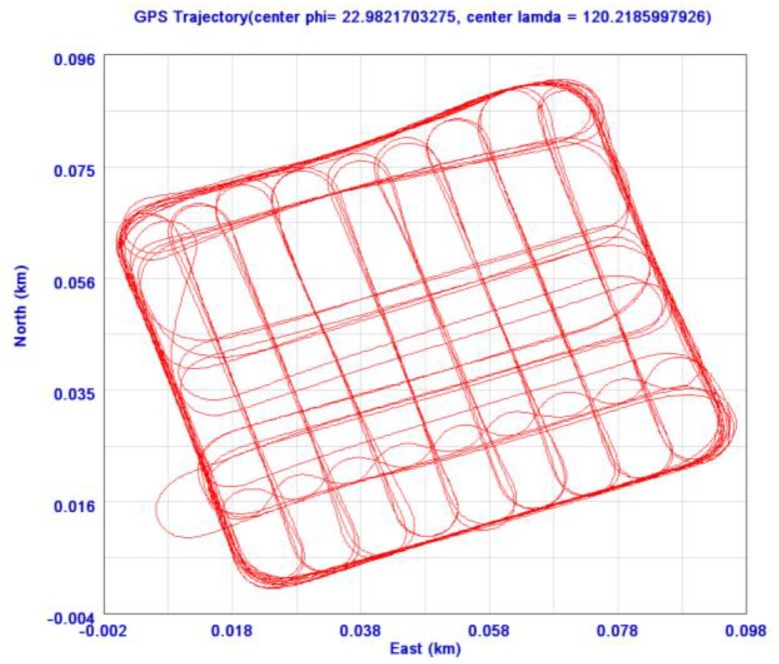
The trajectory of the van test.

The DG positioning error limits the positioning and orientation accuracy of the onboard INS/GPS integrated POS module. Therefore, a land test was conducted to compare the performance of the proposed low cost POS module with a tactical grade INS/GPS system (SPAN-CPT). The GPS measurements provided by both systems were processed using GrafNav™ software (Waypoint Consulting Inc., Calgary, Canada) in carrier phase differential GPS mode to ensure their kinematic positioning accuracy. The reference trajectory was generated by the SPAN-CPT system with dual frequency GPS carrier phase measurements and the test trajectory was generated with the proposed POS module with single frequency GPS carrier phase measurements as shown in Figure 16. The INS/GPS processing engine developed by the Department of Geomatics, National Cheng Kung University was applied to derive smoothed best estimated POS solutions for further analysis. The parameters of the EKF and the smoother applied in this study were well tuned to represent the best achievable navigation accuracy for the IMUs.

**Figure 17.**
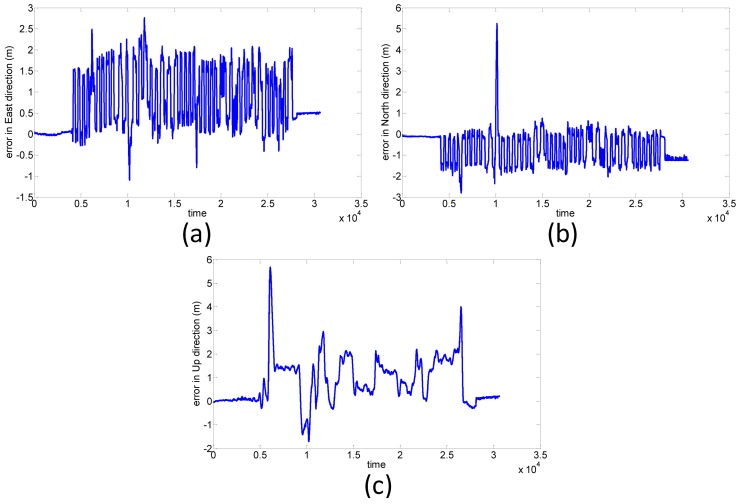
Positional error in (**a**) east; (**b**) north; (**c**) up directions.

**Figure 18.**
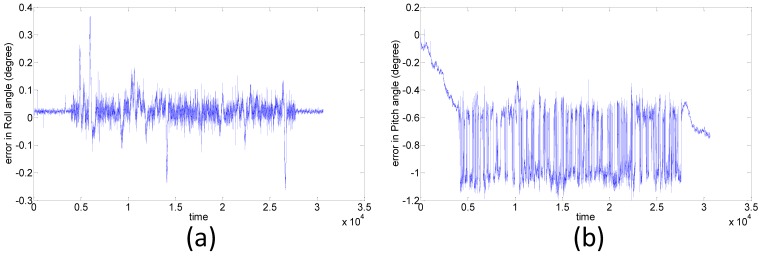

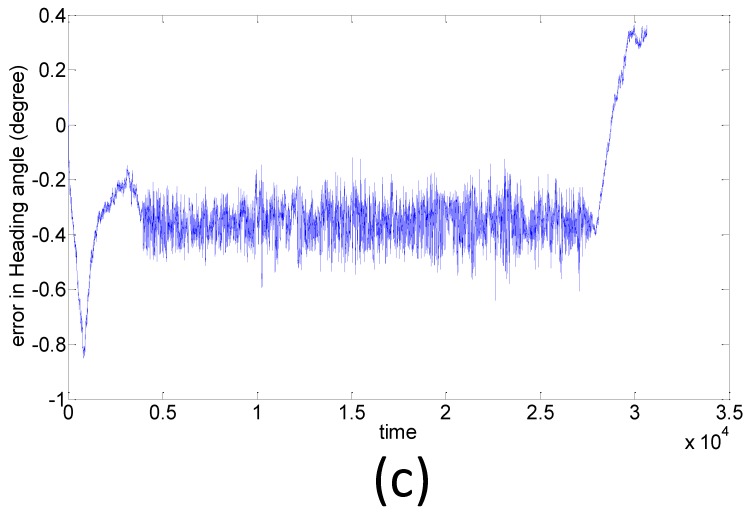
(**a**) Roll; (**b**) Pitch; (**c**) Heading angle errors.

The travel time of the van test is about 50 minutes and this test includes straight lines and sharp turns to simulate a UAV photogrammetry scenario. Figure 17 shows the positional errors of the proposed POS module in the east, north, and up directions, respectively. The preliminary result shows that the RMS errors of the proposed POS module in the east, north and up directions are 0.77, 0.66, 0.83 m, respectively. The RMS errors with roll, pitch and heading angles shown in Figure 18 are 0.02, 0.74, 0.31 degrees, respectively. This experiment verifies that the accuracy of proposed POS module outperforms the traditional SPP mode applied by most commercial photogrammetric UAV platforms.

The approximate error budgets of the proposed system with flight height 300 m above ground are given in Table 7. The error budgets can be further improved using a better IMU, static calibration, and lower flight height. The first phase of this pilot project demonstrates the DG capability of the UAV platform and its accuracy for rapid deployment applications. A second generation tactical grade INS/GPS integrated POS module (gyro bias < 5 deg/h) along with a new UAV platform that can carry more than 50 kg payload is under development to meet conventional mapping standards. In addition, the limited positioning and orientation accuracies provided by the onboard INS/GPS integrated POS module significantly affect the lever arm and boresight angles, resulting in the symmetric errors propagating to DG positioning accuracy. Therefore, instead of using a kinematic calibration procedure, future studies will be conducted to implement a static ground calibration procedure to improve the DG positioning accuracy of the proposed UAV based photogrammetric platform.

The primary contribution of this study is the implementation of a UAV based photogrammetric platform with DG ability and the verification of its performance in terms of DG accuracy. In addition, the preliminary results indicate that the DG accuracy in GCP-free mode can meet the requirements for rapid disaster mapping and relief applications. In the future, a one-step approach will be developed to guarantee accurate lever arms and boresight angle calibrations and a cluster based tightly coupled integrated scheme will be investigated to guarantee the stability of POS solutions. The total cost of the proposed POS module is below 10,000 US dollars thus, making it suitable for rapid disaster relief deployment to provide near real time spatial information. Generally speaking, the data processing time for the DG module, including POS solution generalization, interpolation, EOP generation, and feature point measurements, is less than one hour.

## 5. Conclusions

This study develops a DG based UAV photogrammetric platform where an INS/GPS integrated POS system is implemented to provide the DG capability of the platform. The performance verification indicates that the proposed platform can capture aerial images successfully. The preliminary results illustrate that horizontal DG positioning accuracies in the x and y axes are around 5 m with 300 m flight height. The positioning accuracy in the z axis is less than 10 m. Such accuracy is good for near real time disaster relief. The DG ready function of proposed platform guarantees mapping and positioning capability even in GCP free environments, which is very important for rapid urgent response for disaster relief. Generally speaking, the data processing time for the DG module, including POS solution generalization, interpolation, EOP generation, and feature point measurements, is less than one hour.

In addition, future studies will be conducted to implement a static ground calibration procedure to improve the DG positioning accuracy of the proposed platform. A one-step approach will be developed to guarantee accurate lever arm and boresight angle calibrations and a cluster based tightly coupled integrated scheme will be investigated to guarantee the stability of POS solutions for practical GCP-free applications.

## Figures and Tables

**Table 1.** The comparison of sensor orientation methods.

**Type**		**Observations**	**Control**	**Accuracy**	**Cost**	**Efficiency**
Bundle Block Adjustment In-direct Geo-referencing		Tie points	Lots of GCP	High	GCP collection	Poor
GNSS-assisted AT		Tie points GNSS	Several GCP	High	GCP collection	Medium
Direct sensor orientation (DSO)	GNSS/INS-assisted AT (ISO)	Tie points GNSS INS	A few GCP	High	Minor: GCP collection Major: IMU	High
	Direct Geo-referencing (DG)	GNSS INS	No	Dependent on the used IMU	Dependent on the used IMU	Excellent

**Table 2.** Comparison of various airborne photogrammetric platforms (adopted from [1]).

**Platform**	**System**						**Applications**						
	IMU	GPS	LIDAR	Camera	DG payload weights	Cost	Large area	Small area	Mobility	Weather limitation	DG accuracy (3DRMS)	Cyber city	Rapid Disaster Relief
Fixed wing aircraft	Tactical grade	Geodetic grade	Yes	Digital/film	> 50 kg	High	Yes	Yes	Low	High	< 20 cm	Yes	Low
Helicopter	Tactical grade	Geodetic grade	Yes	Digital	> 50 kg	High	No	Yes	Low	High	< 20 cm	Yes	Low
UAV	MEMS	L1 phase	Yes	Digital	< 25 kg	Low	No	Yes	High	Low	< 50 cm	Yes	High
UAV Helicopter	MEMS	L1 phase	Yes	Digital	< 10 kg	Low	No	Yes	High	Low	< 50 cm	Yes	High

**Table 3.** IOP of EOS 5D Mark II.

Principal distance	c = 20.6478 mm
Principal point offset in x-image coordinate	xp = −0.0819 mm
Principal point offset in y-image coordinate	yp = −0.0792 mm
3^rd^-order term of radial distortion correction	K1 = 2.38021e − 04
5^th^-order term of radial distortion correction	K2 = −4.75072e − 07
7^th^-order term of radial distortion correction	K3 = 5.80760e − 11
Coefficient of decentering distortion	P1 = 1.0121e − 05
Coefficient of decentering distortion	P2 = 2.7671e − 06
No significant differential scaling present	B1 = 0.0000e + 00
No significant non-orthogonality present	B2 = 0.0000e + 00

**Table 4.** Result of lever arm calibration.

	**Lever arm (m)**
X	−0.0242351647
Y	−0.0117635940
Z	0.2297472133
X Std	0.3164630204
Y Std	0.3255619817
Z Std	0.7197633872

**Table 5.** Result of boresight angle calibration.

	**Boresight angle (degree)**
Omega	−0.4303516599
Phi	0.5206395403
Kappa	0.4340006141
Omega Std	1.2816189958
Phi Std	1.0017573973
Kappa Std	1.8544649419

**Table 6.** Results of DG test.

**Check point**	**dx (m)**	**dy (m)**	**dz (m)**
CK00	−4.4282	8.5913	−4.8998
CK02	−0.6265	0.1095	11.7058
CK07	8.0347	4.0368	−3.6966
CK14	5.0358	4.427	2.6559
CK15	−6.0085	0.5407	0.2545
CK17	−2.9827	9.4068	−3.2372
CK19	9.6433	8.845	0.1681
CK21	2.0415	−3.8248	11.858
CK22	−9.737	−1.6082	−9.3202
CK25	6.4878	9.7032	5.3624
CK29	6.3257	−0.8802	−4.5371
CK32	4.5103	9.7274	−6.3125
CK34	2.4402	3.087	−3.6986
CK35	−0.9834	2.4748	−7.2566
CK37	0.8938	2.6153	−10.4813
CK40	3.0999	−3.7624	1.2518
CK41	−6.2964	1.606	−8.0329
CK51	−4.928	−2.002	6.8476
CK52	−6.6467	2.691	−6.8845
CK66	−8.8359	5.4114	−6.64
Average	−0.1480	3.0598	−1.7447
RMS	5.7133	5.3069	6.6695
STD	5.8598	4.4486	6.6045

**Table 7.** Error budgets of the proposed system.

**Error source**	**Magnitude**	**Impact on** ${\delta \text{r}}_{\text{i}}^{\text{m}}$ **(DG error)**
INS/GNSS Positional error	1–2 m	1–2 m
INS/GNSS Orientation error	0.5–1 degree	3–6 m with 300 m flight height
Calibration error ${\delta \text{R}}_{\text{m}}^{\text{b}}$	0.5–1 degree	3–6 m with 300 m flight height
Calibration error δa^b^	0.05–0.1 m	0.5–1 m with 300 m flight height
Synchronization error VδT	1–2 ms	3.6–7.2 cm with 120 km/h flying speed
Synchronization error ωδT	1–2 ms	15–30 cm with 300 m flight height when angular ω equals 30 deg/s
